# Health-Related quality of life and the impact of traditional, complementary and integrative Medicine - an Online - Representative Cross-Sectional survey in Germany

**DOI:** 10.1186/s12889-025-23908-5

**Published:** 2025-08-21

**Authors:** Miriam Ortiz, Manfred Wischnewsky, Michael Jeitler, Benno Brinkhaus, Andreas Michalsen, Rasmus Hoffmann, Christian S. Kessler

**Affiliations:** 1 Institute of Social Medicine, Epidemiology and Health Economics, Charité - Universitätsmedizin Berlin, corporate member of Freie Universität Berlin and Humboldt-Universität zu Berlin, Charitéplatz 1, 10117 Berlin, Germany; 2 Department of Internal Medicine and Nature-Based Therapies, Immanuel Hospital Berlin, Königstr. 63, 14109 Berlin, Germany; 3 Charité Competence Center for Traditional and Integrative Medicine (CCCTIM), Charité – Universitätsmedizin Berlin, corporate member of Freie Universität Berlin and Humboldt-Universität zu Berlin, Charitéplatz 1, 10117 Berlin, Germany; 4 Institute for Sociology, Otto-Friedrich-University Bamberg, Feldkirchenstr. 21, 96052 Bamberg, Germany; 5 Department of Mathematics and Computer Science, Universität Bremen, Bibliothekstraße 5, 28359 Bremen, Germany; 6 Institute for General Practice and Interprofessional Care, University Hospital Tuebingen, Tuebingen, Germany; 7 Robert Bosch Center for Integrative Medicine and Health, Bosch Health Campus, Stuttgart, Germany

**Keywords:** Health related quality of life, Traditional medicine, Complementary medicine, Integrative medicine, EQ-5D-5L, Cross-sectional study

## Abstract

**Background:**

Traditional, Complementary, and Integrative Medicine (TCIM) is widely used in Germany. Approximately 70% of the German population report lifetime use, with 32% indicating usage within the past year and 18% currently. This study examines the association between TCIM utilization and health-related factors, including sociodemographic characteristics and health-related quality of life (HRQoL) in Germany.

**Methods:**

An online survey of 4,065 German adults (aged 18–75) was conducted in 2022. HRQoL was assessed with globally recognized and extensively validated instruments using the EQ-5D-5 L descriptive system and index values. We compared HRQoL based on age, gender, income, TCIM use and attitudes, medical conditions, dietary patterns, and social milieus (Sinus-Milieus^®^). The EQ-5D-5 L index ranges from 1 (perfect health) to ≤ 0 (death), EQ VAS from 100 (best imaginable health) to 0 (worst imaginable health).

**Results:**

The mean ± standard error and median (interquartile range) were 68.2 ± 0.4 and 73.0 (32.5) for EQ VAS, and 0.85 ± 0.00 and 0.92 (0.18) for the EQ-5D-5 L index. Lower HRQoL was observed among women, older adults, and individuals with lower socioeconomic status. TCIM users within the past 12 months (31.8%) had significantly lower EQ-5D-5 L index scores (0.82 ± 0.01; 0.90 [0.17]) than non-users (0.87 ± 0.00; 0.94 [0.17]; *p* < 0.001) and reported a significantly higher disease burden (88.0% vs. 68.6%). Musculoskeletal disorders were the most common condition (35.8%), associated with an EQ-5D-5 L index of 0.71 ± 0.01 and EQ VAS of 56.9 ± 0.6. Neurological conditions were linked to the lowest HRQoL, whereas allergies were associated with the highest. Dietary patterns significantly influenced HRQoL, with pescatarians reporting the highest and raw foodists the lowest scores. Among social milieus, the “Precarious Milieu” had the lowest EQ-5D-5 L index (median 0.86 [0.28]), while the “Performer Milieu” had the highest (0.97 [0.11]).

**Conclusion:**

The findings underscore the influence of sociodemographic and socioeconomic determinants on HRQoL and highlight health disparities across social groups. The inverse association between TCIM use and HRQoL may reflect a higher burden of chronic conditions and unmet healthcare needs among TCIM users. Further research is warranted to investigate causal relationships and the role of TCIM in addressing complex health challenges.

**Trial registration:**

ClinicalTrials.gov (NCT05530720).

**Supplementary Information:**

The online version contains supplementary material available at 10.1186/s12889-025-23908-5.

## Background

Traditional, Complementary, and Integrative Medicine (TCIM) is defined by the use of evidence-based methods from complementary medicine including traditional medical whole systems in combination with conventional medicine for the care and health of a whole person [1]. In Germany, “Naturheilkunde” is particularly well known. It can be considered as Traditional European Medicine (TEM) and part of TCIM and comprises of the key elements hydrotherapy, phytotherapy, nutrition, physical exercise and mind-body approaches for health prevention and therapy. Its history traces back among other to the healing tradition of the famous German lay healer Sebastian Kneipp from the 19th century [2]. Today the German healthcare system offers TCIM in the context of health promotion and prevention. Several rehabilitation clinics, some specialized public and private clinics offer a TCIM driven treatment concept. However, in the medical context TCIM is mainly present in the outpatient sector whereby the costs of treatment are borne by the patients themselves. Only single individual services, such as acupuncture for chronic lower back pain or osteoarthritis of the knee can be reimbursed by the statutory health insurance. Physicians have the opportunity to acquire an additional specialist qualification in TEM and acupuncture. TCIM is often part of the medical teaching curriculum and present at universities in research. There are several appointed professors with working groups to foster the scientific evaluation of TCIM across the country [3, 4].

Various studies have shown in the past that a large proportion of the population knows about and uses TCIM in Germany [5, 6]. The results of our recent large-scale online cross-sectional study involving 4,065 participants are in line with these findings. They showed that almost 70% of the participants from this online-representative population used TCIM, 32% within the past year, and 18% currently, mostly due to musculoskeletal pain [4]. In contrast to former studies, this study firstly assessed the Sinus Milieus ^®^. Sinus Milieus^®^ is a social classification system and a tool for societal analyses that groups individuals with similar values, lifestyles, and social positions into groups of “like-minded people” resulting in 10 different milieus. This internationally recognized model (applicable in over 50 countries) allows for a more nuanced understanding of participant backgrounds. The Sinus Milieus^®^ categories are dynamic, reflecting fluidity within social structures. The validated and theory-based model was developed by the Sinus Institute [7]. It includes sociocultural as well as socioeconomic factors and can be used for understanding differences between health outcomes in different social milieus by extensive characterizations of the Sinus Milieus^®^de. The different milieus are described with the attributes “Precarious” milieu, which can be assigned to the lower social class, the “Traditional”, the “Nostalgic Middle Class”, the “Adaptive-Pragmatic Middle Class”, the “Consumer – Hedonistic” and the “Neo-Ecological” milieus which are more likely to find in the middle class and further the “Conservative Upscale”, the Post – Materialistic”, the “Performer” and the “Expeditive” milieus, which are assigned to the upper social class. Fundamental value orientations are taken into account as well as everyday attitudes (to work, family, leisure, consumption, media, etc.) and the social situation to identify the individual Sinus Milieu. The Sinus-Milieus therefore take a holistic view of people and the reference system of their living environment [8].

Also in contrast to former studies, this study firstly utilized a generic instrument to assess health-related quality of life (HRQoL).

Key aspects of HRQoL include physical functioning, which refers to the ability to carry out daily activities such as walking, dressing, and bathing. Emotional well-being encompasses mood, anxiety levels, and overall mental health. Social functioning relates to the ability to maintain relationships and engage in social activities, while cognitive functioning includes memory, attention, and decision-making abilities.

The EQ-5D-5 L is a widely used and validated standardized, non-disease-specific questionnaire developed by the EuroQol Group for measuring HRQoL across diverse populations and health conditions [9]. The EQ-5D-5 L comprises a descriptive system and the EQ Visual Analogue Scale (EQ VAS) [10–12].

While HRQoL has been the subject of numerous studies in Germany, its relationship to TCIM, alongside sociodemographic characteristics and other potentially health-related factors such as diseases and dietary patterns, remains unexplored. To date, no national study has investigated the association between TCIM use and HRQoL using EQ-5D-5 L in conjunction with Sinus Milieus classification. The aim of this study was to investigate the relationship between TCIM, health-related factors, including sociodemographic characteristics and HRQoL in Germany.

## Methods

### Study design and participants

A cross-sectional online survey was conducted in the fall of 2022 among the German-speaking population aged 18–75 years residing in Germany [4]. The study aimed to assess the use and acceptance of TCIM, including its impact on HRQoL. The Ethics Committee of the Charité Universitätsmedizin Berlin approved the study (EA2/128/22), the study was registered on ClinicalTrials.gov (NCT05530720).

### Sampling and recruitment

Details of the study were published in [4]. In summary, to ensure a sample representative of the online population, the survey was conducted by three established German market research institutes: Conversio, Sinus Institute, and Respondi Institute. Participants were recruited through Respondi’s online access panel using a quota-based sampling approach. This method ensured balanced representation across key demographic characteristics, including age, gender, education level, and geographic region. The quota structure was designed to align with the methodology of the best4planning (B4P) study, which is recognized for its representative sampling based on a randomized selection of over 30,000 individuals. Additionally, quota control measures were implemented to minimize potential socio-demographic discrepancies between the study sample and the general population [13].

### Survey instrument

A comprehensive questionnaire was used consisting of 94 items. It covered a broad range of topics: sociodemographics, use of TCIM, attitudes toward TCIM, diagnoses for which TCIM were used, importance and familiarity with terms used in this context, the role of TCIM in the context of the Covid-19 pandemic, nutrition, Ayurveda, attitude and behavior toward TCIM, Sinus milieu indicator and the EQ-5D-5 L health related quality of life questionnaire [4].

### Overall health status

There is frequent interest in employing a single question, such as “How would you rate your overall health status?“, to assess health status within health surveys. An individual’s self-assessment of their health status as excellent, very good, good, fair, or poor serves as a widely used summary indicator of health status (it is e.g. the first question in the SF-36 questionnaire [14]).

### EQ-5D-5 L index and EQ VAS: health profiles, overall self-rated health status and results from the EQ-5D-5 L index

The EQ-5D-5 L descriptive system of health-related quality of life states consists of five dimensions (mobility, self-care, usual activities, pain/discomfort, anxiety/depression). Each dimension is divided into five levels of severity (no problems (1); slight problems (2); moderate problems (3); severe problems (4); extreme/unable to problems (5)). A respondent’s health state or profile is represented by a five-digit code, where each digit corresponds to a severity level on one of the five dimensions.

An EQ-5D-5 L index of a patient is derived by applying a formula that attaches country-specific index values (weights) to each of the levels in each dimension. A collection of weights is called an EQ-5D-5 L value set. The EQ-5D-5 L value sets are determined for each country based on population-representative data. We used the standard EQ-5D-5 L value set for Germany [15].

The second part consists of a visual analogue scale (EQ VAS score) that allows individuals to rate their overall health on a scale of 0 (worst imaginable health) to 100 (best imaginable health).

By combining the EQ-5D-5 L index and EQ VAS, EQ-5D-5 L provides a comprehensive assessment of health-related quality of life [15, 16].

### Statistical analysis

For continuous variables, we report means and standard deviations (SD) if normally distributed, and medians and interquartile ranges (IQR) otherwise. For reporting the skewed EQ VAS and EQ-5D-5 L data we reported in addition mean and standard errors of mean (SEM) for direct comparisons with legacy studies. Categorical variables are presented as frequencies and percentages. Normality was assessed using the Kolmogorov-Smirnov test. Differences in categorical variables were analyzed with the Pearson’s χ2 test. Crosstab analysis revealed patterns, correlations, and trends among categorical variables. The independent-samples Kruskal-Wallis test or the independent-samples median test were used to compare a non-normally distributed continuous variable across more than two groups. Dunn`s test was employed as a post-hoc test for Kruskal-Wallis. When additional covariates and factors were included in the model with non-normally distributed continuous variables we used Quade’s nonparametric ANCOVA as a specialized General Linear Model. An alternative approach would be “aligned ranks transformation ANOVA” (ART ANOVA), which allows multiple independent variables, interactions, and repeated measures (R-library ARTool). To obtain Quade’s nonparametric ANCOVA we ranked both the dependent variables (EQ VAS, EQ-5D-5 L index) and the covariate (age). A linear regression was performed, regressing the ranks of the dependent variable on the ranks of the covariates and saving the unstandardized residuals (ignoring the grouping factor). In the final step, an univariate General Linear Model (GLM) was conducted, using the unstandardized residuals from the previous linear regression as the dependent variable and the grouping variable as factor [17–19]. The F-test resulting from this GLM is the nonparametric Quade-test. Optimal cut off value for EQ VAS and EQ-5D-5 L index were calculated with receiver operating curves at maximum Youden index. Additionally, we used the Exhaustive Chi-squared Automatic Interaction Detector decision tree algorithm to predict a target variable based on the data. Statistical significance was set at *p* < 0.05. All statistical analyses were performed using R version 4.3 and IBM SPSS Statistics version 30.

## Results

### Basic characteristics

4,065 participants aged 18–75 were included in this cross-sectional study. 51.7% were female. Mean age was 49.3 ± 15.8 and median age 51.0 years. Educational attainment was distributed as follows: 42.5% with higher education (university degree or equivalent) and 56.8% with primary or secondary education. Approximately half (54.3%) reported a net household income between €2,000 and €5,000, with 7.5% earning above and 38.2% below this range. For detailed sociodemographic characteristics, please refer to the main publication [4]. Among the 1,291 participants (31.8%) who used TCIM within the past year, female participants were slightly overrepresented (60.7%), and the majority (59.1%) had a monthly household income between 2,000€ and 5,000€. Educational attainment was higher compared to the overall sample, with 39.1% holding a university degree or equivalent. Interest in basic medical knowledge was significantly higher among TCIM users (25.9%) compared to non-users or those unaware of TCIM use (11.9% and 15.3%, respectively; *p* < 0.001). (Table 1)

**Table 1:** Basic characteristics with EQ VAS and EQ-5D-5 L index

	HRQoL							
	All (*n* = 4065)		EQ-VAS		*p*-value	EQ-5D-5 L		*p*-value
	n	%						
*Gender*			Mean ± SEM	median (IQR)		Mean ± SEM	Median (IQR)	
Male	1947	47.9	68.9 ± 0.51	75.0 (31)	0.042*	0.87 ± 0.004	0.94 (0.17)	< 0.001
Female	2101	51.7	67.5 ± 0.48	71.0 (33)		0.84 ± 0.005	0.91 (0.17)	
Diverse	17	0.4	67.0 ± 5.1	72.0 (33)		0.86 ± 0.044	0.91 (0.13)	
*Age in years*								
18 to24	273	6.7	72.9 ± 1.22	77.0 (30)	< 0.001	0.88 ± 0.01	0.94 (0.17)	< 0.001
25 to 34	643	15.8	72.3 ± 0.85	79.0 (28)		0.90 ± 0.01	0.94 (0.13)	
35 to 44	672	16.5	72.3 ± 0.81	77.0 (28)		0.90 ± 0.01	0.94 (0.14)	
45 to 54	723	17.8	66.9 ± 0.87	71.0 (34)		0.84 ± 0.01	0.92 (0.19)	
55 to 64	888	21.8	64.3 ± 0.77	69.0 (32)		0.81 ± 0.01	0.90 (0.19)	
65 to 75	866	21.3	65.8 ± 0.75	70.0 (32)		0.83 ± 0.01	0.91 (0.17)	
*Education*								
No general school-leaving certificate (yet), still a pupil at a general school	30	0.7	65.4 ± 4.4	63.5 (36)	< 0.001	0.81 ± 0.05	0.91 (0.16)	< 0.001
Primary (elementary, basic) school leaving certificate without completed apprenticeship/vocational training	251	6.2	58.7 ± 1.5	60.0 (36)		0.78 ± 0.02	0.87 (0.23)	
Primary school leaving certificate with completed apprenticeship/vocational training	885	21.8	63.6 ± 0.8	68.0 (34)		0.81 ± 0.01	0.89 (0.20)	
Secondary school without A-levels (German: Realschulabschluss/Mittlere Reife/Oberschule) or equivalent qualification	1173	28.9	67.9 ± 0.7	73.0 (32)		0.85 ± 0.01	0.92 (0.18)	
A-levels, (technical) university entrance qualification without studies	751	18.5	71.6 ± 0.7	77.0 (26)		0.88 ± 0.01	0.94 (0.15)	
Studies (university, college, university of applied sciences, polytechnic)	949	23.3	72.4 ± 0.7	78.0 (25)		0.89 ± 0.01	0.94 (0.14)	
PhD	26	0.6	80.5 ± 2.8	85.0 (20)		0.90 ± 0.04	0.94 (0.11)	
*Personal monthly net income*								
No own income	175	4.3	64.8 ± 1.9	70.0 (32)	< 0.001	0.83 ± 0.02	0.91 (0.23)	< 0.001
Up to 1000 €	887	21.8	63.4 ± 0.8	68.0 (32)		0.81 ± 0.01	0.89 (0.21)	
1000–2000 €	1549	38.1	66.6 ± 0.6	70.0 (34)		0.83 ± 0.01	0.91 (0.71)	
2000–3000 €	959	23.6	72.1 ± 0.7	78.0 (28)		0.89 ± 0.01	0.94 (0.14)	
3000–4000 €	311	7.7	75.8 ± 1.0	80.0 (20)		0.92 ± 0.01	0.94 (0.11)	
4000–5000 €	99	2.4	70.8 ± 2.0	75.0 (26)		0.89 ± 0.02	0.94 (0.13)	
> 5000 €	85	2.1	78.8 ± 2.1	85.0 (20)		0.92 ± 0.01	1.0 (0.11)	
*Net monthly household income*								
Up to 1000 €	505	12.4	59.8 ± 1.1	60.0 (36)	< 0.001	0.79 ± 0.01	0.87 (0.25)	< 0.001
1000–2000 €	1047	25.8	63.9 ± 0.7	68.0 (32)		0.81 ± 0.01	0.89 (0.20)	
2000–3000 €	1049	25.8	69.2 ± 0.7	75.0 (28)		0.86 ± 0.01	0.92 (0.17)	
3000–4000 €	735	18.1	71.9 ± 0.7	76.0 (24)		0.88 ± 0.01	0.94 (0.14)	
4000–5000 €	424	10.4	73.7 ± 1.0	79.0 (24)		0.90 ± 0.01	0.94 (0.12)	
> 5000 €	305	7.5	76.9 ± 1.2	83.0 (20)		0.91 ± 0.01	0.97 (0.12)	
*Sinus Milieus* ^®^								
Conservative Upscale Milieu	429	10.6	68.3 ± 1.1	73.0 (33)	< 0.001	0.85 ± 0.01	0.92 (0.18)	< 0.001
Post-Materialist Milieu	575	14.1	71.4 ± 0.9	77.0 (28)		0.88 ± 0.01	0.94 (0.14)	
Performer Milieu	452	11.1	77.2 ± 0.9	81.0 (20)		0.91 ± 0.01	0.97 (0.11)	
Expeditive Milieu	417	10.3	71.2 ± 1.1	76.0 (27)		0.88 ± 0.01	0.94 (0.15)	
Adaptive-pragmatic middle class Milieu	475	11.7	68.4 ± 1.0	73.0 (31)		0.86 ± 0.01	0.92 (0.19)	
Nostalgic middle class Milieu	443	10.9	63.2 ± 1.1	66.0 (33)		0.81 ± 0.01	0.91 (0.20)	
Traditional Milieu	289	7.1	65.5 ± 1.3	70.0 (33)		0.86 ± 0.01	0.91 (0.16)	
Precarious Milieu	346	8.5	57.6 ± 1.3	60.0 (37)		0.75 ± 0.01	0.86 (0.28)	
Consumer-Hedonistic Milieu	314	7.7	65.5 ± 1.3	70.0 (33)		0.84 ± 0.01	0.92 (0.22)	
Neo-Ecological Milieu	325	8.0	68.8 ± 1.2	73.0 (27)		0.85 ± 0.01	0.92 (0.17)	

SEM (standard error of mean); IQR (interquartile range)

* female vs. male; female vs. male vs. diverse: *p* = 0.124

### Overall health status

The single question “How would you rate your overall health status?” demonstrates a strong correlation with other indicators of health status. In this study, 32.5% of participants rated their overall health status as excellent or very good, 45.3% considered it good, and 22.2% reported it as fair or poor. Among participants younger than 50 years, a higher proportion (45.4%) assessed their health as excellent or very good, while only 13.2% rated it as fair or poor. In contrast, only 20.3% of participants aged 50 years and older reported excellent or very good health status, with 30.8% indicating fair or poor health.

Among participants over 50 years old living in a ‘precarious milieu’, the percentage reporting excellent or very good health decreased to 10.5%, while the percentage reporting fair or poor health increased to 51.9%. In contrast to this result, participants under 50 years with a high school diploma who belonged to the ‘performer milieu’ according to the Sinus Milieus^®^ had the most favorable health outcomes. Health was estimated to be excellent or very good by 68.6% of the sample, while only 1.4% indicated less good or bad health.

### EQ-5D-5 L health States

**Fig. 1:**
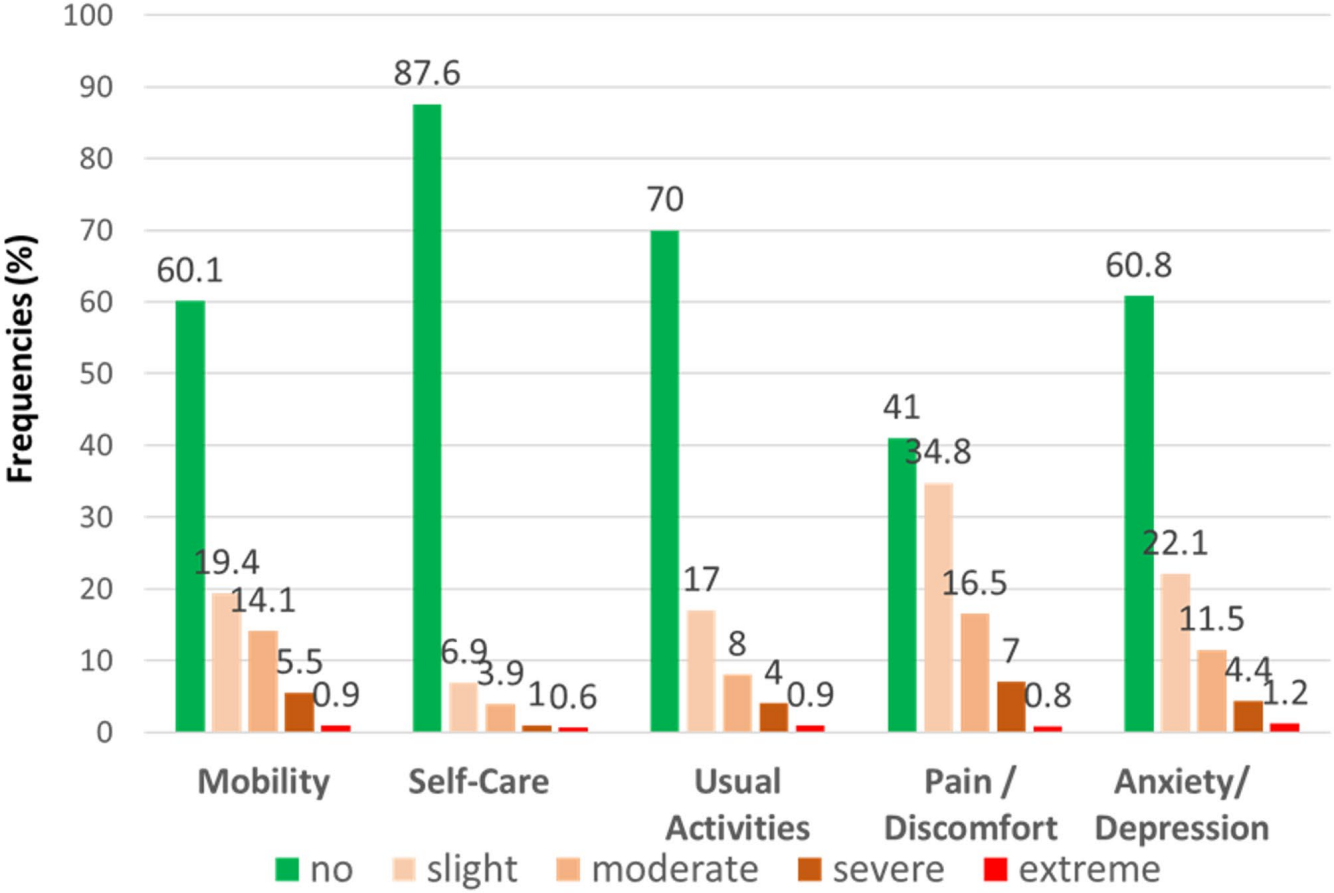
EQ-5D-5 L frequencies (%) reported by dimension and level

Figure 1 illustrates the proportions (%) of reported level 1 (no) to level 5 (extreme/unable to) problems for each of the 5 EQ-5D-5 L dimensions. The EQ-5D-5 L dimension with the lowest prevalence of reported problems was “self-care”. Only 12.4% of participants reported at least one health problem (level 2 or higher). The two dimensions with the highest prevalence of impairment were anxiety/depression, affecting 39.2%, and “pain/discomfort,” affecting 59.0% of participants (Fig. 1). 28.9% of the study population reported no problems across the various dimensions of health (health state 11111). Within this group, 53.7% were female. 39.9% of participants without any reported problems considered TCIM to be very or rather important, 31.8% used TCIM within the last 12 months, and 41.4% consumed dietary supplements, vitamin preparations, or herbal products daily. Extreme problems in any dimension were infrequent, ranging from 0.6% (self-care) to 1.2% (anxiety/depression).

**Fig. 2:**
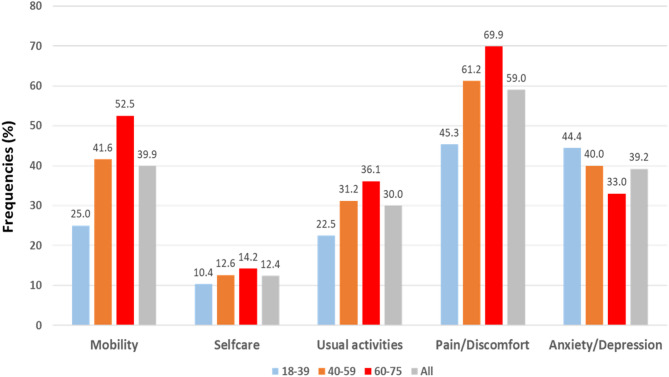
Reported problems by five dimensions (% of any problem) across three distinct age groups

Due to the typically low prevalence of severe or extreme problems in general population surveys, it is sometimes more convenient to dichotomise the EQ-5D-5 L levels into ‘no problems’ (level 1) and ‘any problems’ (levels 2, 3, 4 and 5), thereby changing the profile into frequencies of reported problems. In a pooled dataset, Fig. 2 depicts the frequencies (%) of any problems for each of the 5 EQ-5D-5L dimensions across three distinct age groups. Significant differences (*p* < 0.001) were observed in the percentages of any problems between the three age categories for each dimension. Problems with mobility exhibited the most pronounced increase from 25.0–52.5% with advancing age, while problems with anxiety/depression interestingly decreased from 44.4–33.0% with age. Across all age groups, the proportion of problems with pain/discomfort due to pain was consistently higher than the proportion of problems in the other dimensions.

### EQ VAS

EQ VAS was not normally distributed (Kolmogorov-Smirnov test: asymptotic sig. (2-sided), *p* < 0.001; Suppl. Figure 1). The mean ± SEM and median (IQR) of EQ VAS for all participants were 68.2 ± 0.4 and 73.0 (32.5). Females had a significant lower score than men (females: 67.5 ± 0.5, 71.0 (33.0); males: 68.9 ± 0.5, 75.0 (31.0); Bonferroni adj. *p* = 0.015). Median EQ VAS exhibited a significant inverse relationship with age and a significant positive association with both education level and net monthly household income. Participants with a PhD demonstrated the highest median EQ VAS (85.0 [21]), while those with no general school leaving certificate had the lowest median EQ VAS (63.5 [37]). Furthermore, EQ VAS scores exhibited a highly significant dependence on total income, with scores significantly lower for individuals with a total income of up to 1000€ (median EQ VAS 60.0 [37]) compared to those with an income exceeding 5000€ (EQ VAS 83.0 [21]) (Table 1).

**Fig. 3:**
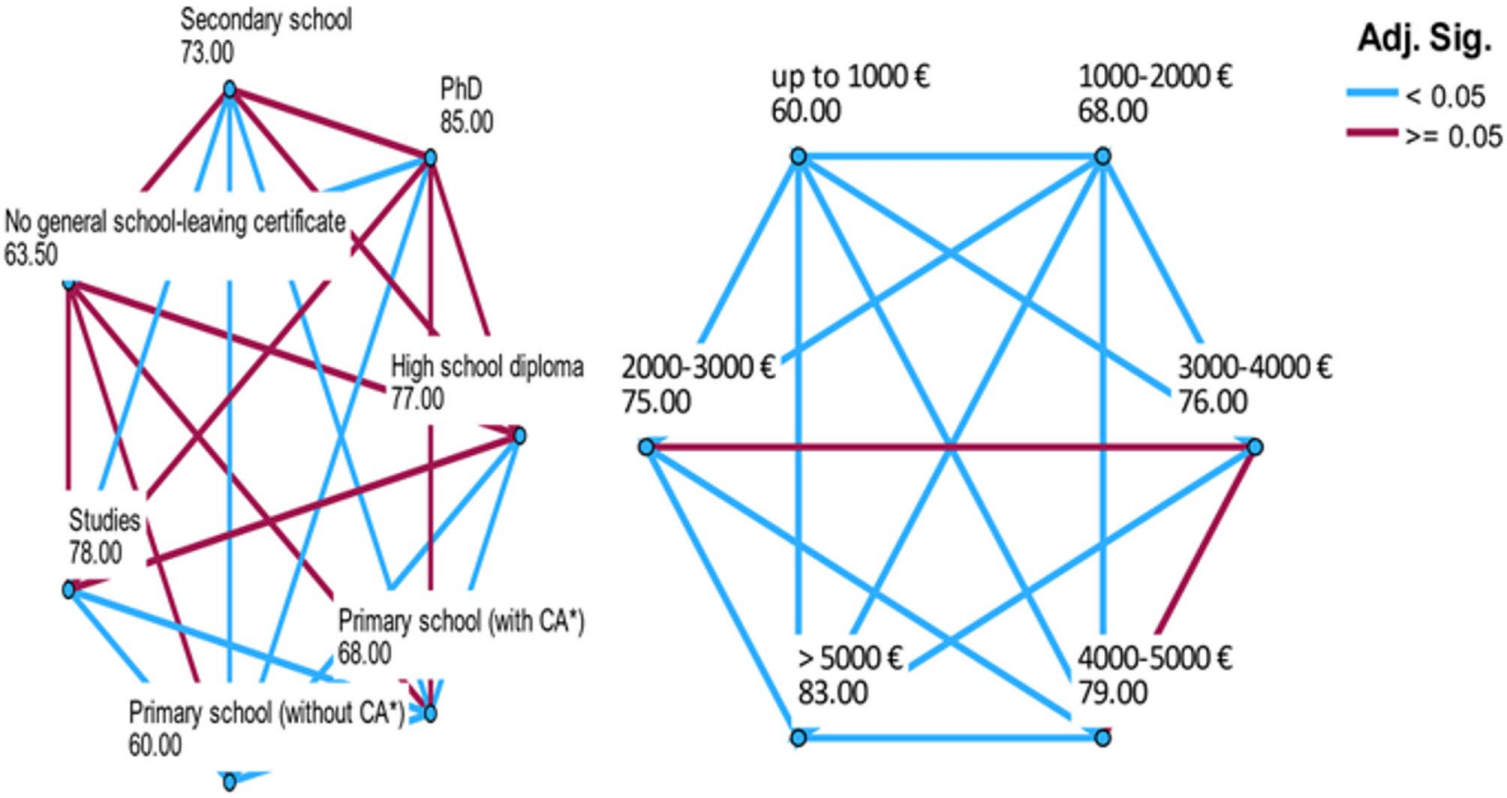
EQ VAS: Pairwise comparisons of the various levels of education **a** and net monthly household income **b** (blue line: significant differences, red line: no significant differences)

All pairwise comparisons of median EQ VAS scores across different education levels and net monthly household income categories except for two are statistically significant Fig. 3.

**Fig. 4:**
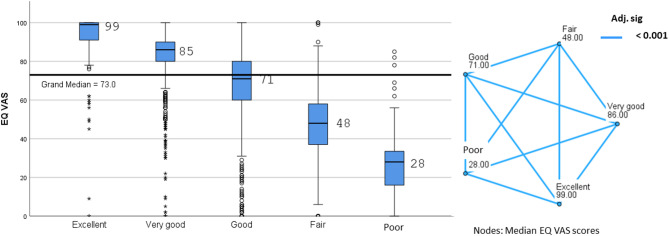
Independent-samples median test of EQ VAS and pairwise comparison across overall health status categories

Next, we demonstrate that overall health status exhibits a strong correlation with EQ VAS scores. Median EQ VAS scores decrease as overall health status deteriorates (Fig. 4, a). All pairwise comparisons between the health status categories are statistically highly significant (Fig. 4, b).

### EQ-5D-5 L index

EQ-5D-5 L index was also not normally distributed (Kolmogorov-Smirnov test: *p* < 0.001; Suppl. Figure 2). The mean ± SEM and median (IQR) of EQ-5D-5 L index for all participants were 0.85 ± 0.00 and 0.92 (0.18). Overall, male individuals appear to have a significantly (*p* < 0.001) better health-related quality of life (median EQ-5D-5 L index: 0.94 (0.17)) than female (EQ-5D-5 L index : 0.91 (0.17)) (Table 1).

The median EQ-5D-5 L index values stratified by self-reported general health status gave again insights into the meaning of the index within our sample. We observed mean ± SEM and median index values (IQR) of 0.97 ± 0.01 and 1.0 (0.00) for excellent, 0.95 ± 0.00 and 1.0 (0.06) for very good, 0.89 ± 0.00 and 0.92 (0.11) for good, 0.67 ± 0.01 and 0.75 (0.32) for fair and 0.35 ± 0.03 and 0.29 (0.47) for poor overall health status (Suppl. Figure 3, a). Larger differences in index values were observed between health status categories as perceived overall health status worsened. The differences between any of two overall health status categories were highly significant (Suppl. Figure 3, b).

### Optimal cut off values for EQ VAS and EQ-5D-5 L index

**Fig. 5:**
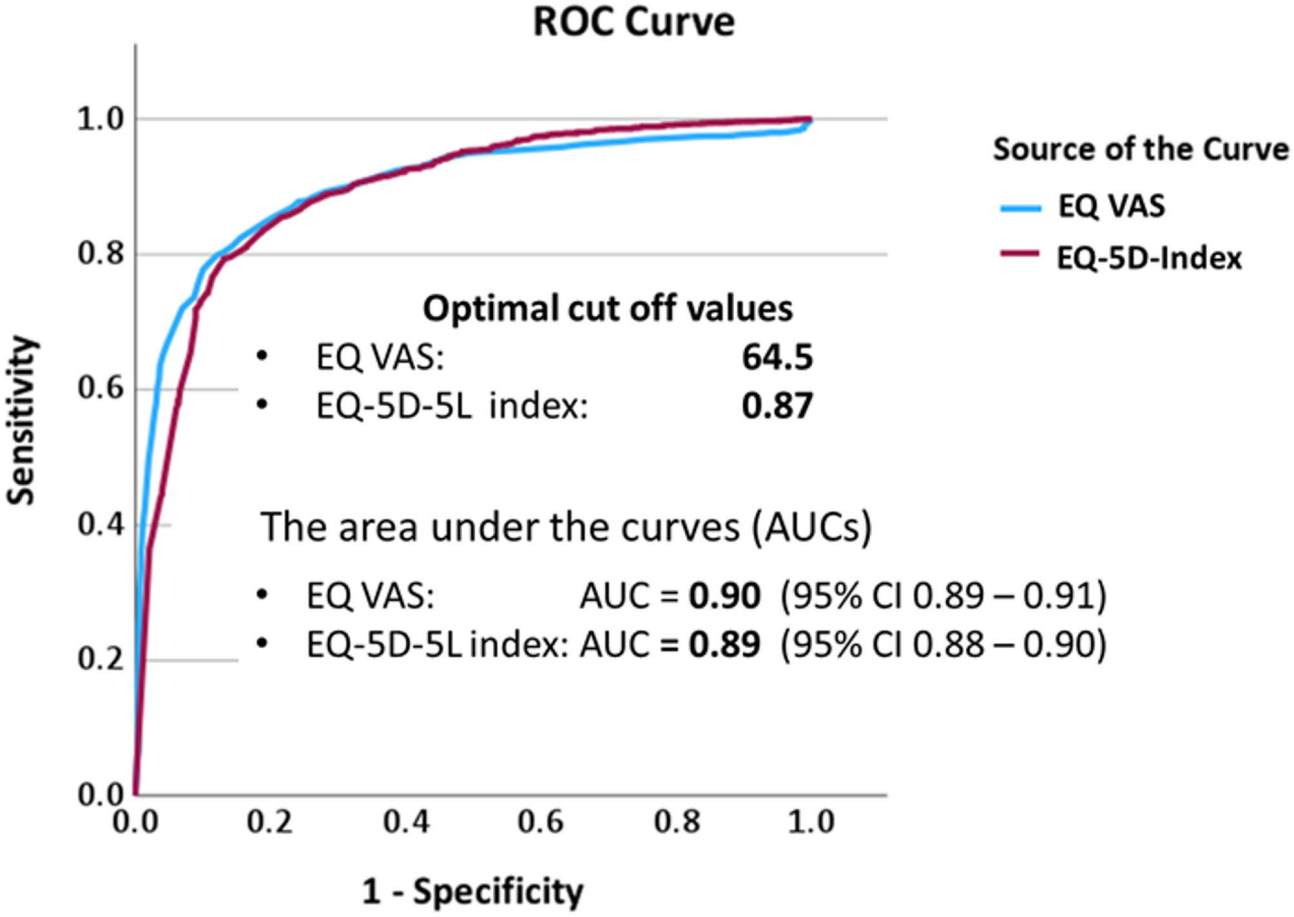
Optimal cut off values for EQ VAS and EQ-5D-5 L index with corresponding areas under the curves

In order to calculate optimal cut off values for EQ VAS and EQ-5D-5 L index values we transform the 5 overall health status categories into two categories, utilizing the categories “good to excellent” and “fair to poor”. The optimal cut off value for EQ VAS, statistically defined as the best compromise between sensitivity and specificity, is 64.5 at maximum Youden index 0.678 (identically to the maximum Kolmogorov-Smirnov (K-S) metric). The area (AUC) under the receiver operating characteristic (ROC) curve is 0.90 (std error 0.005; 95% CI 0.89–0.91; *p* < 0.001) (Fig. 5). The optimal cut-off value for the EQ-5D-5 L index is 0.87 at maximum Youden index 0.662. The AUC is 0.89 (std error 0.006; 95% CI 0.88–0.90; *p* < 0.001) (Fig. 5). The AUCs for EQ VAS and the EQ-5D-5 L index were not significantly different. This indicates that both methods exhibit comparable performance in determining cut-off values for classifying individuals with good to excellent health from those with moderate to poor health.

### TCIM use, attitude towards TCIM and disease burden

The mean ± SEM and median (IQR) EQ-5D-5 L index values of the study population, who have used TCIM within the last 12 months (*n* = 1291, 31.8%), was 0.82 ± 0.01 and 0.90 (0.17). The mean value 0.82 is below the optimal cut off value 0.87. The medians of the EQ-5D-5 L index across the attitudes towards TCIM are not equal (asymptotic sig. *p* < 0.001) nor across the attitudes towards conventional medicine (asymptotic sig. *p* = 0.013). All pairwise comparisons (independent samples median test) for attitude towards TCIM are not significant (*p* > 0.05) except for three (very positive (0.92) – undecided (0.97), *p* = 0.017; mostly positive (0.91) – undecided (0.97), *p* = 0.008; and mostly positive (0.91) – neutral (0.92), *p* = 0.030). All pairwise comparisons for attitude towards conventional medicine are not significant (*p* > 0.05) except one (the difference ´very positive´ (0.94) and ´mostly positive´ (0.92) was significant (*p* = 0.033)). Conversely the attitude towards TCIM depends significantly (*p* < 0.001) on the EQ-5D-5 L index (Suppl. Figure 4).

The highest percentage of positive attitudes towards TCIM was 57.2% for respondents with an EQ-5D-5 L index between 0.78 and 0.94. The lowest percentage of positive attitudes towards TCIM was 45.9% for respondents with EQ-5D-5 L index > 0.94. Similar results were found for attitudes towards conventional medicine, which also depended significantly on the EQ-5D-5 L index (*p* < 0.001). A clear pattern emerged: positive attitudes towards conventional medicine were consistently higher than those towards TCIM, with an average difference of 10% or more. The most positive attitudes towards conventional medicine (66.5%) were observed among participants with EQ-5D-5 L index values between 0.78 and 0.94 (Suppl. Figure 4).

### Medical conditions

We subsequently investigated EQ VAS and EQ-5D-5 L index in relation to various medical conditions (Table 2). Neurological diseases were associated with the lowest HRQoL (EQ VAS 53.6 ± 1.7, 51.0 [36], EQ-5D-5 L-index 0.69 ± 0.02, 0.79 (0.35)), while allergies (EQ VAS 66.6 ± 0.7, 70.0 [31], EQ-5D-5 L index 0.82 ± 0.01, 0.91 (0.19)) were linked to the highest HRQoL.

**Table 2:** Percent of responses, percent of cases, EQ VAS and EQ-5D-5 L index of patients with various current diseases or diseases in the past (multiple responses)

Diseases	Responses		Percent of cases^b^	EQ-VAS^c^		EQ-5D-5 L-Index^c^	
	*N*	Percent		Mean ± SEM	Median (IQR)	Mean ± SEM	Median (IQR)
Neurological diseases (e.g. stroke, dementia such as Alzheimer’s and Parkinson’s, multiple sclerosis)	172	2.1%	5.7%	53.6 ± 1.7	51.0 (35)	0.69 ± 0.02	0.79 (0.35)
Chronic gastrointestinal diseases (e.g. irritable bowel syndrome, ulcerative colitis, Crohn’s disease)	280	3.4%	9.2%	56.2 ± 1.3	58.0 (32)	0.71 ± 0.02	0.84 (0.39)
Painful disorders of the musculoskeletal system (e.g. chronic back pain, osteoarthritis, rheumatoid arthritis)	1085	13.1%	35.8%	56.9 ± 0.6	60.0 (32)	0.71 ± 0.01	0.82 (0.33)
Mental illnesses (e.g. depression, anxiety disorder, burnout)	820	9.9%	27.0%	56.4 ± 0.7	58.0 (32)	0.71 ± 0.01	0.81 (0.29)
Chronic respiratory diseases (e.g. bronchial asthma, COPD, bronchitis)	548	6.6%	18.1%	57.9 ± 0.9	60.0 (35)	0.73 ± 0.01	0.84 (0.31)
Diabetes mellitus	425	5.1%	14.0%	58.6 ± 1.1	60.0 (33)	0.76 ± 0.01	0.85 (0.22)
Cancer (e.g. breast cancer, prostate cancer, colon cancer, lung cancer)	165	2.0%	5.4%	56.5 ± 1.7	58.0 (35)	0.77 ± 0.02	0.86 (0.26)
Thyroid diseases (e.g. hyperthyroidism, hypothyroidism)	587	7.1%	19.4%	61.6 ± 0.9	65.0 (32)	0.77 ± 0.01	0.87 (0.23)
Cardiovascular diseases (e.g. high blood pressure, heart failure, arteriosclerosis, cardiac arrhythmias)	936	11.3%	30.9%	60.5 ± 0.7	64.0 (32)	0.78 ± 0.01	0.87 (0.21)
Headache disorders (e.g. migraines, tension headaches)	761	9.2%	25.1%	63.5 ± 0.8	67.0 (30)	0.79 ± 0.01	0.88 (0.19)
Acute respiratory illnesses (e.g. acute respiratory tract infection)	558	6.7%	18.4%	63.7 ± 0.92	68.0 (30)	0.80 ± 0.01	0.89 (0.18)
Acute gastrointestinal illnesses (e.g. acute infection of the gastrointestinal tract)	417	5.0%	13.7%	63.4 ± 1.1	68.0 (32)	0.79 ± 0.01	0.89 (80.21)
Skin diseases (e.g. neurodermatitis, psoriasis, acne)	521	6.3%	17.2%	64.2 ± 1.0	69.0 (32)	0.79 ± 0.01	0.88 (0.18)
Allergies (e.g. hay fever, house dust mite allergy, animal hair allergy, food allergy)	1032	12.4%	34.0%	66.6 ± 0.68	70.0 (30)	0.82 ± 0.01	0.91 (0.19)
Total	8307	100.0%	273.9%	64.4 ± 0.4	69.0 (31)	0.82 ± 0.00	0.89 (0.16)

^a^Dichotomy group tabulated at value 1 (yes)

^b^Percent of cases = number of diseases/number of patients with any of these diseases *100. Number of patients with any of these diseases = 3041

^c^mean ± SEM (SEM: standard error of mean), median (interquartile range) of all patients with any of these diseases

Among the 1291 respondents who used TCIM within the last 12 months, 88.9% reported having current or past diseases from the aforementioned list. In contrast, 69.4% of non-users reported having any of these diseases. This difference suggests a higher disease burden among TCIM users. This finding may explain the significantly lower EQ VAS and EQ-5D-5 L index observed among respondents who used TCIM within the last 12 months compared to non-users.

### HRQoL and dietary patterns

82.7% of the respondents were omnivorous or flexitarians, 4.0% lacto-ovo vegetarians, 2.3% pescatarians and only 1.5% vegan. Within this study (Table 3), individuals following a raw food diet reported the lowest HRQoL (EQ VAS 57.1 ± 5.1, 57 [37]; EQ-5D-5 L index 0.70 ± 0.06 0.81 (0.45) whereas pescatarians reported the highest (EQ VAS 70.6 ± 2.2, 74 [36]; EQ-5D-5 L index 0.86 ± 0.02, 0.92 (0.20)). EQ VAS and EQ-5D-5 L index depended significantly on dietary patterns.

**Table 3:** Frequency, mean, median and interquartile range of EQ VAS and EQ-5D-5 L index for various dietary patterns

Dietary patterns	Frequency (%)	EQ-VAS			EQ-5D-5 L-index		
		Mean ± SEM	Median (IQR)	Sig.*	Mean ± SEM	Median (IQR)	Sig.*
Mixed food [omnivorous]	2199 (54.1)	69.0 ± 0.5	74 (29)	*p* = 0.022	0.86 ± 0.00	0.92 (0.17)	*p* < 0.001
Mixed food, with few animal products [flexitarian]	1161 (28.6)	68.3 ± 0.6	73 (33)		0.84 ± 0.01	0.91 (0.19)	
Vegetarian, including dairy products and eggs [lacto-ovo-vegetarian]	161 (4.0)	66.0 ± 1.9	70 (31)		0.84 ± 0.02	0.91 (0.21)	
Plant-based – almost no animal foods (max. 5%)	109 (2.7)	66.0 ± 2.1	69 (29)		0.83 ± 0.02	0.91 (0.22)	
Vegetarian plus fish [pescatarian]	95 (2.3)	70.6 ± 2.2	74 (35)		0.86 ± 0.02	0.92 (0.20)	
Vegan, without animal products	61 (1.5)	68.4 ± 3.1	73 (24)		0.85 ± 0.03	0.91 (0.13)	
Raw food	20 (0.5)	57.1 ± 5.1	57 (36)		0.70 ± 0.06	0.81 (0.45)	
Other	77 (1.9)	66.2 ± 2.8	75 (34)		0.82 ± 0.03	0.92 (0.23)	
I don’t know	182 (4.5)	62.6 ± 2.0	60 (38)		0.840.02	0.94 (0.22)	

* Independent-Samples Kruskal-Wallis Test

### Multivariate analysis (Quade’s nonparametric ANOVA): dietary patterns, age, gender, EQ VAS and EQ-5D-5 L index

Quade’s nonparametric ANOVA, with age as a covariate and dietary patterns and gender as factors, reinforces the previous findings. Between-subjects effects analyses revealed significant corrected models for both EQ VAS (*p* < 0.001) and EQ-5D-5 L index (*p* < 0.001). Dietary patterns significantly impacted HRQoL (EQ VAS *p* = 0.016; EQ-5D-5 L index *p* = 0.007) with observed power of 89.3% for EQ VAS and 93.0% for EQ-5D-5 L index, while gender did not show a significant effect in these models (EQ VAS *p* = 0.416; EQ-5D-5 L index *p* = 0.060). No significant interaction was observed between dietary patterns and gender (*p* = 0.296). Pairwise comparisons demonstrated significant differences between certain dietary groups, such as omnivores and raw foodists (EQ VAS *p* = 0.022; EQ-5D-5 L index *p* < 0.001) and pescatarians and raw foodists (EQ VAS *p* = 0.044; EQ-5D-5 L index *p* = 0.010). Suppl. Figure 5 illustrates the estimated marginal means of unstandardized residuals from Quade’s nonparametric ANOVA.

### Milieu-specific differences in EQ-5D-5 L index values

**Fig. 6:**
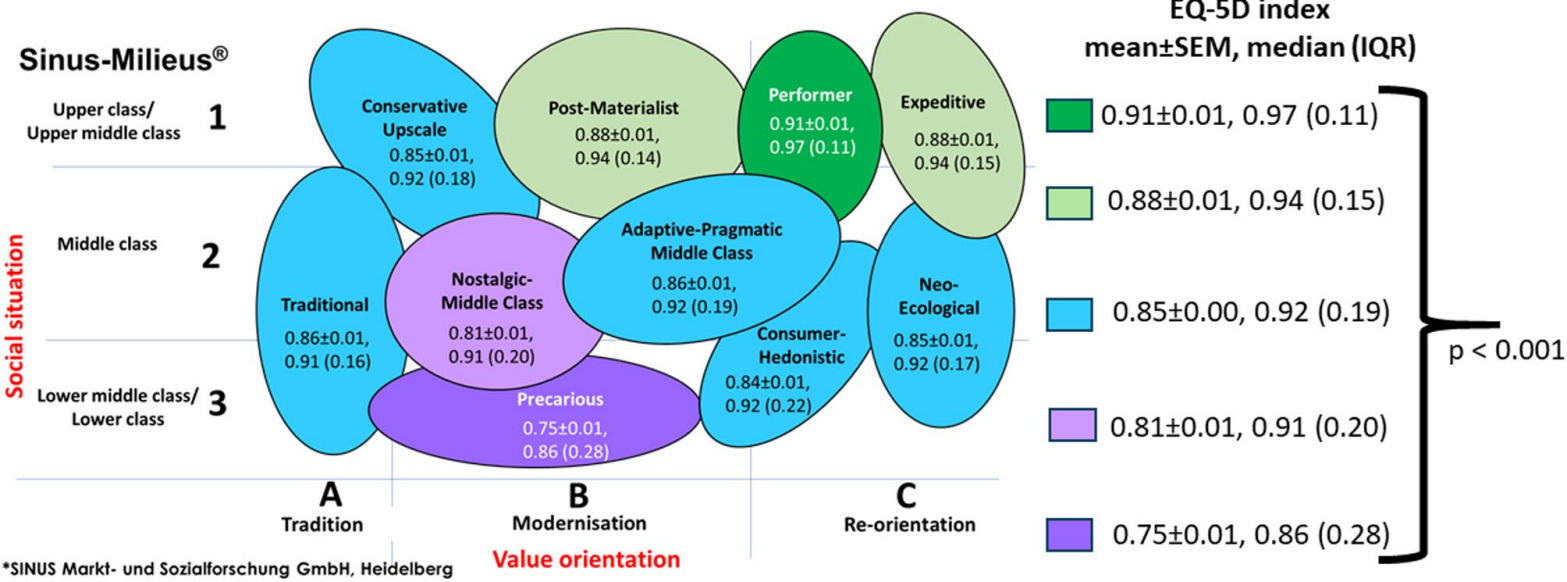
Mean ± SEM and median (interquartile range) EQ-5D-5 L index values across Sinus Milieus^®^

The Sinus models all have a very similar graphic structure. The vertical axis shows the social situation (ranging from low to high) and the horizontal axis shows the value orientation (ranging from traditional to postmodern). The higher a milieu is located in this graph, the more upscale its education, income and occupational group; the further to the right it extends, the more modern in a sociocultural sense the basic orientation of the respective milieu. There were significant milieu-specific differences for EQ VAS and EQ-5D-5 L index (Table 1). Figure 6 shows the graphical structure with mean ± SEM, median EQ-5D-5 L index values and the corresponding IQR.

The EQ-5D-5 L index was found to be highly significantly associated with Sinus milieus^®^ (*p* < 0.001). The ´Precarious Milieu´ exhibited the lowest median EQ-5D-5 L index (0.86 (0.28), *n* = 346, 9.5%), while the ´Performer Milieu´ demonstrated the highest (0.97 (0.11), *n* = 452, 11.1%). No significant differences were found between milieus with the same color category.

## Discussion

Study results presented revealed that while overall HRQoL measured by the EQ-5D-5 L in our sample was generally good, several factors were associated with lower HRQoL: increasing age, female gender, lower income, belonging to the “Precarious Milieu” (as defined by the Sinus^®^ Milieu model), neurological and pain diseases, the use of TCIM. Conversely, pescatarian dietary patterns were linked to higher HRQoL.

The study population exhibited demographic characteristics typical of the general German population, with a slight female overrepresentation [20]. Notably, TCIM users differed significantly from the overall sample in terms of education and income, suggesting that these sociodemographic factors may influence the adoption of TCIM [4]. This observation aligns with previous research demonstrating a higher prevalence of TCIM use among female individuals with higher socioeconomic status [5, 6].

Our EQ-5D-5 L data generally aligns with findings from a previous German study by Grochtdreis et al. [16]. While our study showed slightly lower mean and median EQ-5D-5 L index values and VAS, both studies identified similar determinants of HRQoL, such as age, gender and higher education resp. income. The association between age and EQ VAS is consistent with prior research, which indicates that older individuals tend to report lower HRQoL, potentially due to an increasing disease burden with advancing age [21].

In our study, male reported slightly higher HRQoL compared to female participants. Interestingly, when analyzing HRQoL across age categories, we observed that the gender gap tended to decrease with increasing age. This suggests that gender-specific role distributions and societal stereotypes, particularly the “double burden” faced by female persons during their reproductive years (combining career and child-rearing responsibilities) [22, 23], may play a role. It’s important to note that while the quality of life for female persons in Germany has generally improved in recent decades, this improvement has not been uniform across all population groups [24]. Furthermore, a systematic review and meta-analysis by Zhou consistently found lower EQ-5D-5 L index values for female patients across a broad spectrum of diseases [25].

The observed association between income and EQ VAS underscores the well-established socioeconomic gradient in health [26, 27]. The relationship between health literacy and socioeconomic status may also play a significant role in this correlation [28].

The relationship between HRQoL and the use and attitudes toward TCIM and conventional medicine is intriguing. It seems contradictory that a higher socioeconomic status was related to a higher HRQoL and a higher use of TCIM while TCIM users showed a lower HRQoL compared to non-users. TCIM user characteristics may explain this contradiction: While this study was not designed to establish causality, we hypothesize, that individuals with a higher burden of disease (e.g., multiple or more severe health conditions) resulting in lower HRQoL may be more inclined to explore non-conventional treatment options. We see this often in clinical practice [29]. This possible effect of a higher disease burden among TCIM users aligns with findings but also from previous studies on TCIM utilization [30, 31]. Kemppainen et al., analyzing data from the European Social Survey Round 7, found that only 9% of individuals with no health problems used TCIM in the past 12 months, compared to more than 20% of those with various medical conditions. The use of TCIM by individuals with reduced HRQoL may reflect their demand for holistic and self-directed treatment approaches, as many TCIM interventions involve self-application. If we assume that TCIM use is influenced by the type and number of diseases and reflects unmet treatment needs, this finding strongly supports the need for better promotion of the coexistence and cooperation between conventional and TCIM, particularly for this patient group. However, we have no information about ”conventional“ medicine use in this survey thus we cannot demonstrate the correlation of any therapeutic use other than TCIM. The female gender as a characteristic of TCIM users may also explain the observed lower HRQoL. Our study indicated that female gender was associated with reduced HRQoL and a greater burden of disease. Understanding the reasons for the higher prevalence of TCIM use among women and their specific usage patterns (preventive or therapeutic) warrants further investigation. Beyond medical conditions, several other factors may contribute to TCIM use. A recent qualitative study highlighted the influence of national health systems, available treatment options, communication skills between patients and practitioners, treatment expectations, and health beliefs held by both patients and practitioners in different European countries [32].

The EQ-5D-5L results indicate significant variations in HRQoL across different medical conditions. Pain/discomfort, followed by mobility restrictions, were the most prevalent health issues impacting HRQoL, consistent with findings from other population-based studies. Pain, particularly low back pain, is a major contributor to work absenteeism worldwide [33]. The Global Burden of Disease working group identified chronic low back pain as a leading cause of disability-adjusted life years (DALYs) in Northern Europe [34]. Individuals with neurological diseases exhibited the lowest EQ-5D-5L index values. This finding is generally consistent with the results of a systematic review by Zhou et al. on EQ-5D-5L indices in different chronic conditions [25]. However, it is important to note that the review focused on specific conditions (e.g., multiple sclerosis, Parkinson’s disease, spinal cord injury), whereas our study analyzed a broader category of “neurological diseases”. Interestingly, allergic diseases were associated with the highest EQ-5D-5L index values. This finding may be attributed to the timepoint of data collection, which occurred during the fall and winter seasons. Individuals with seasonal allergic rhinitis, which is highly prevalent, may experience fewer symptoms during these months.

The observed differences in HRQoL between dietary patterns corroborate the well-established link between nutrition and health. More than 60% of our study participants considered a healthy diet to be important for their health. They showed higher EQ-5D-5 L index values than those with a more neutral attitude towards nutrition [35]. Diets rich in fiber and fish, such as the Mediterranean diet, are believed to play a crucial role in healthy aging and quality of life [36]. Extensive research has demonstrated a strong correlation between dietary patterns and HRQoL. Diets rich in fruits, vegetables, whole grains, lean proteins, and healthy fats are associated with significantly higher HRQoL, while diets high in processed foods, unhealthy fats, and excessive sugar tend to correlate with lower HRQoL [37]. Diets including fish, such as the Mediterranean Diet, are prevalent in “Blue Zones” such as Japan (Okinawa), Greece (Ikaria island), Italy (Sardinia), and Costa Rica (Nicoya), and are often associated with increased longevity [38]. In our study, pescatarians reported higher HRQoL compared to individuals following raw food diets, further emphasizing the general consensus on the benefits of a balanced diet. However, the lack of significant differences between pescatarians and omnivores is most likely the result of the widely varying case numbers, but needs further investigation.

The Sinus Milieu^®^ model provides valuable insights into the sociocultural context of HRQoL. The association between EQ-5D-5 L index and different milieus highlights the significant influence of lifestyle, values, and social factors on health outcomes. Sinus Milieus^®^ are used to explore the impact of sociocultural aspects on attitudes, development, and other relevant factors. While limited research has examined the correlation between treatment preferences in medicine and social milieus, a recent study by Kleineberg-Massuthe et al. demonstrated the relevance of the sociocultural milieu on disease severity and treatment access in patients with psychosomatic diseases, with individuals from the “Precarious Milieu” experiencing more severe disease burden [39].

### Future research

Future research should explore the mid to long-term effects of TCIM use for therapy or prevention on HRQoL vice versa for example in observational and clinical studies, investigate the underlying mechanisms linking dietary patterns to health outcomes, and delve deeper into the relationship between sociocultural factors and health disparities. This requires the implementation of adequate outcome measures in clinical studies. The fact that a higher socioeconomic status goes in line with better health and higher TCIM use but better health status is associated with lower TCIM use seems contradictory and use warrants further research.

### Strength and limitations

To our knowledge, this study is the first to comprehensively evaluate the use and attitudes toward complementary and integrative medicine (TCIM), alongside health-related quality of life (HRQoL). We assessed HRQoL using the well-established EQ-5D-5 L questionnaire, within a population-representative sample in Germany. The data set provides additional information to the main publication on socioeconomic and -cultural aspects as well as medical data and conditions in the context of HRQoL. The study also demonstrates how the EQ-5D-5 L results can be used and interpreted in a methodologically correct manner in a cross-sectional study. It is essential to acknowledge the limitations of this study, such as the cross-sectional design, which precludes causal inferences. Additionally, the reliance on self-reported data may introduce biases including a possible gender bias in the estimation of the own health status. Further strength and limitations of this online cross-sectional study e.g. on data assessment and response rate are given elsewhere [4].

## Conclusion

The findings underscore the influence of sociodemographic and socioeconomic determinants on HRQoL and highlight health disparities across social groups. The inverse association between TCIM use and HRQoL may reflect a higher burden of chronic conditions and unmet healthcare needs among TCIM users. Further research is warranted to investigate causal relationships and the role of TCIM in addressing complex health challenges.

## Electronic supplementary material

Below is the link to the electronic supplementary material.

Supplementary Material 1

Supplementary Material 2

Supplementary Material 3

Supplementary Material 4

Supplementary Material 5

Supplementary Material 6

## Data Availability

The datasets used and/or analysed during the current study are available from the corresponding author on reasonable request.

## Abbreviations

ANCOVA: Analysis of covariance
ANOVA: Analysis of variance
EQ-5D-5 L: European quality of life − 5 dimensions-5 Levels questionnaire
GLM: General linear models
HRQoL: Health related quality of life
IQR: Interquartile range
TCIM: Traditional complementary and integrative medicine
VAS: Visual analogue scale
