# Supplementary figures and images for: Health-Related quality of life and the impact of traditional, complementary and integrative Medicine - an Online - Representative Cross-Sectional survey in Germany
Source: BMC Public Health. 2025 Aug 21;25:2870. doi: 10.1186/s12889-025-23908-5 (PMC12369224; doi:10.1186/s12889-025-23908-5)

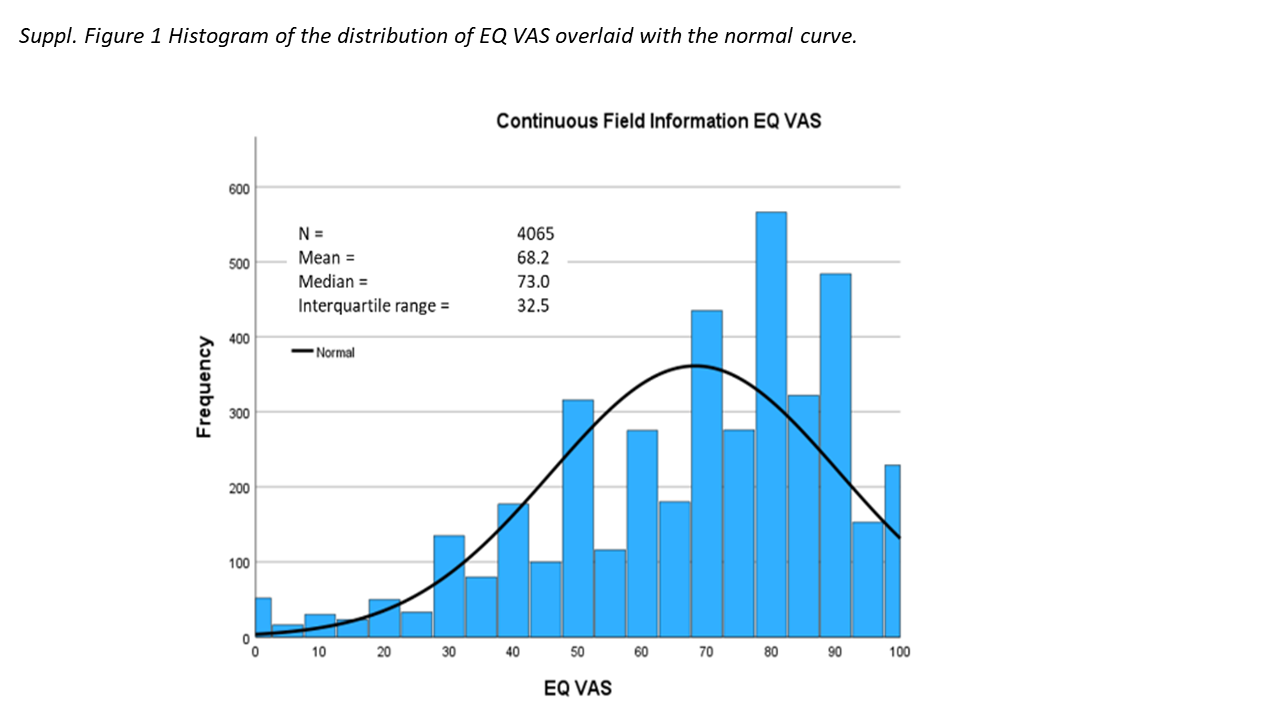

Supplement: Supplementary file 2 — Supplementary Material 2 [file 12889_2025_23908_MOESM2_ESM.tif]

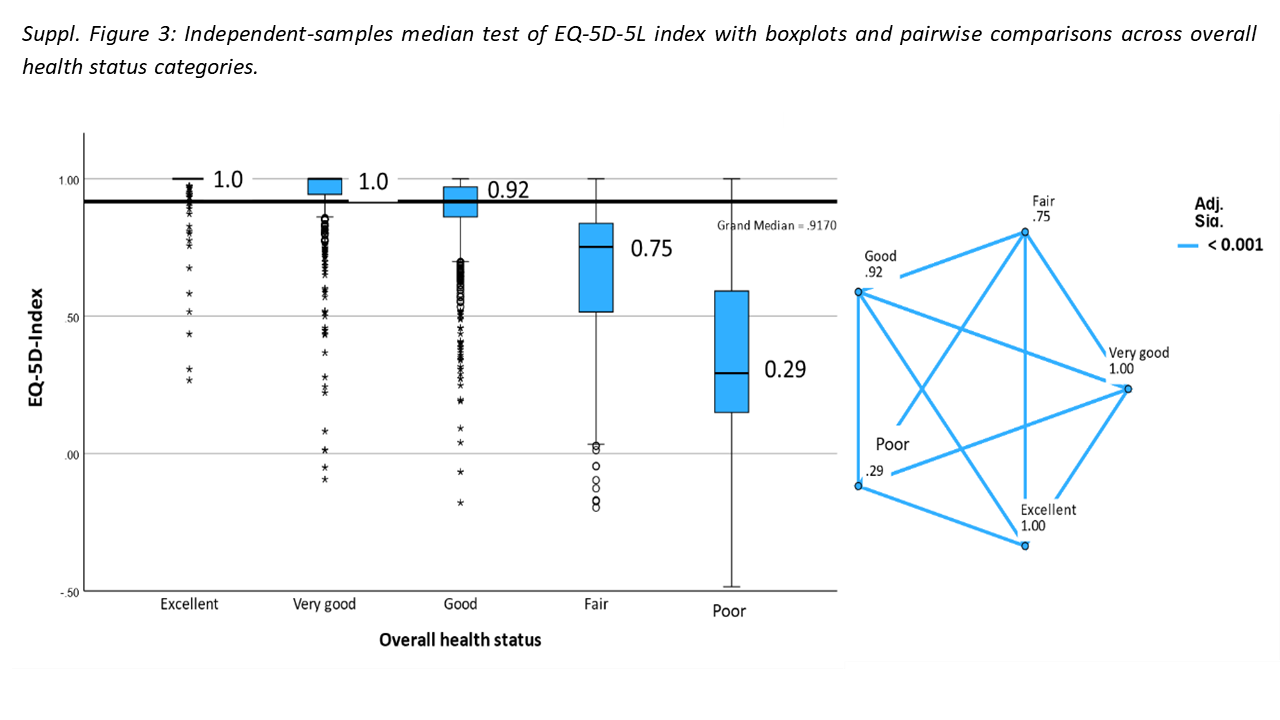

Supplement: Supplementary file 3 — Supplementary Material 3 [file 12889_2025_23908_MOESM3_ESM.tif]

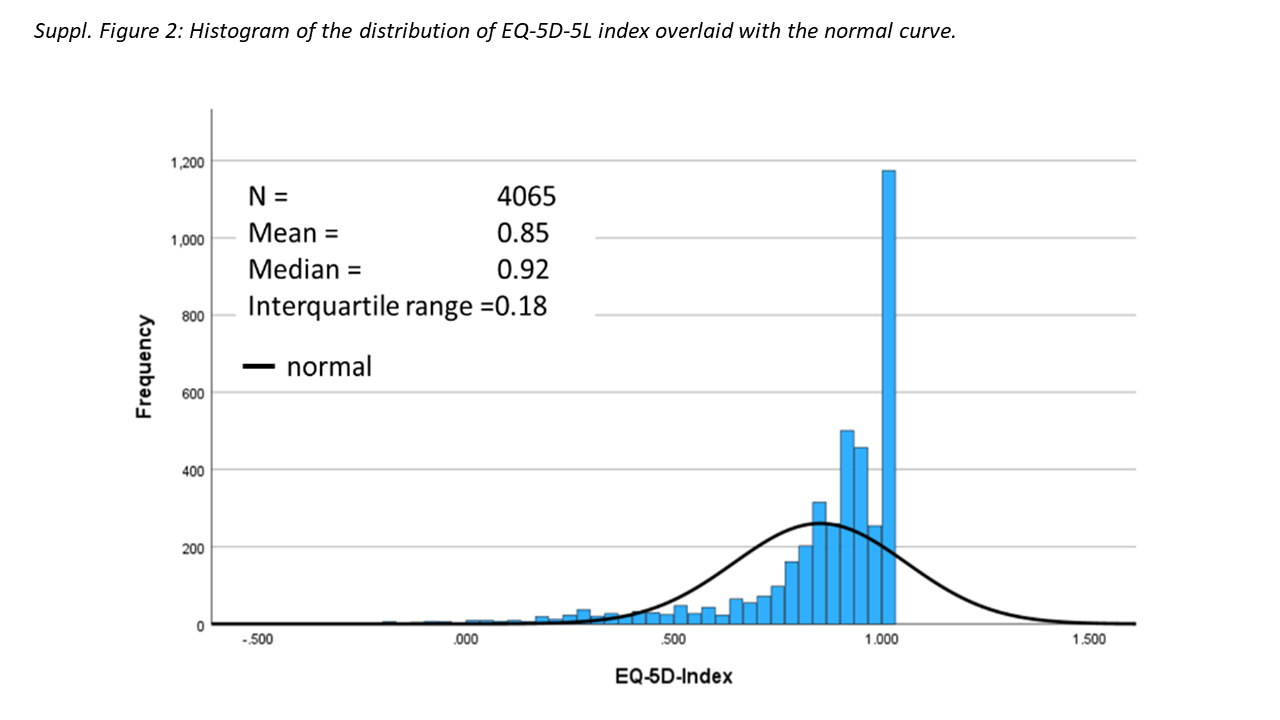

Supplement: Supplementary file 4 — Supplementary Material 4 [file 12889_2025_23908_MOESM4_ESM.tif]

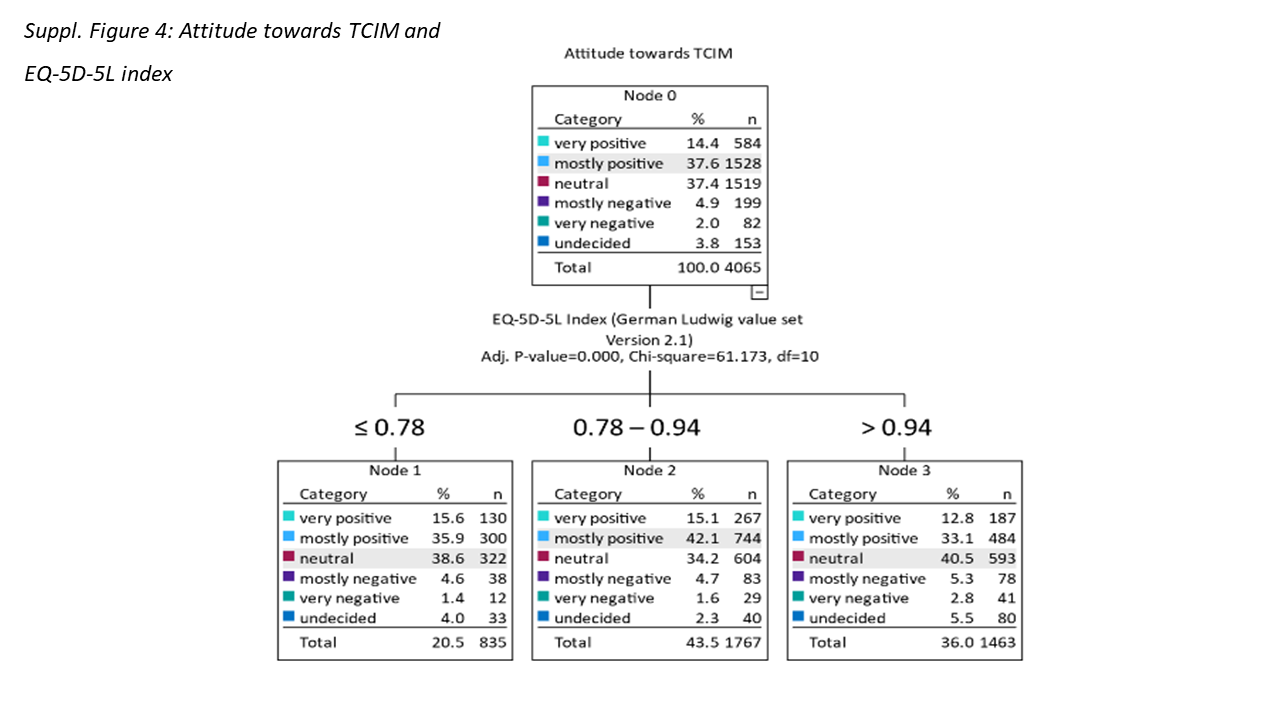

Supplement: Supplementary file 5 — Supplementary Material 5 [file 12889_2025_23908_MOESM5_ESM.tif]

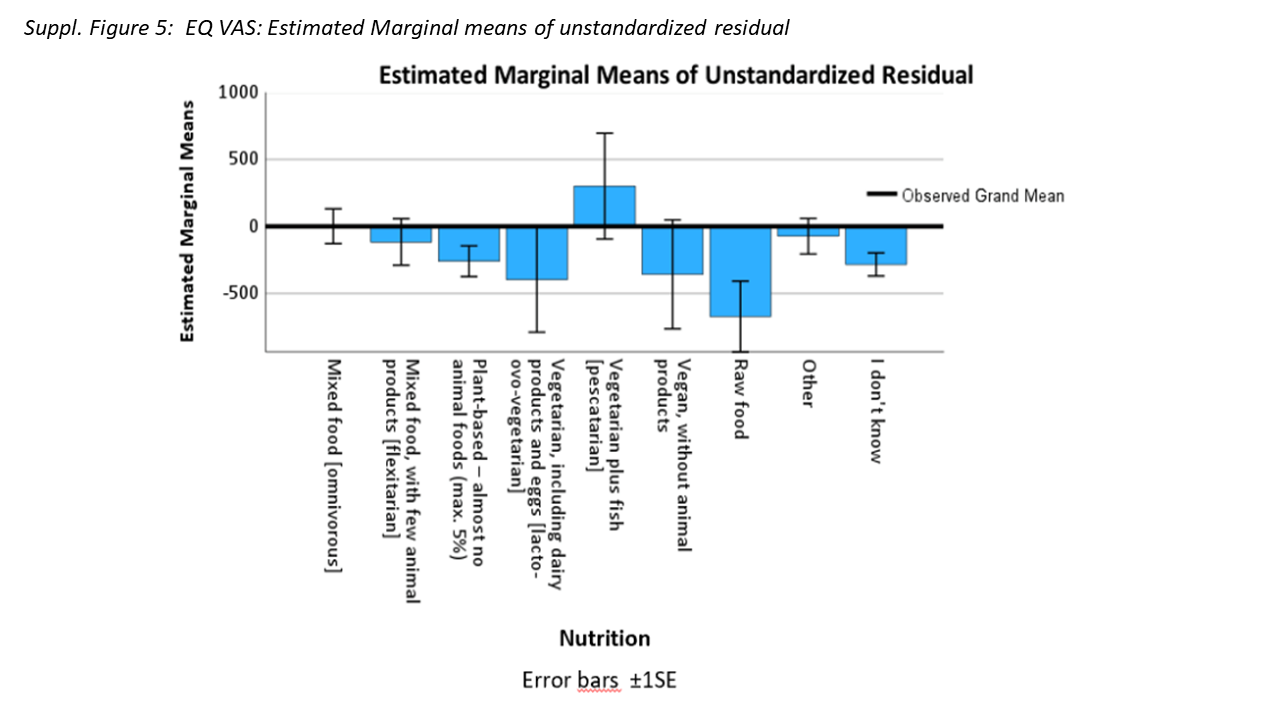

Supplement: Supplementary file 6 — Supplementary Material 6 [file 12889_2025_23908_MOESM6_ESM.tif]
